# *BIRC2–BIRC3* amplification: a potentially druggable feature of a subset of head and neck cancers in patients with Fanconi anemia

**DOI:** 10.1038/s41598-021-04042-9

**Published:** 2022-01-07

**Authors:** Khashayar Roohollahi, Yvonne de Jong, Govind Pai, Mohamad Amr Zaini, Klaas de Lint, Daoud Sie, Martin A. Rooimans, Davy Rockx, Elizabeth E. Hoskins, Najim Ameziane, Rob Wolthuis, Hans Joenje, Susanne I. Wells, Josephine Dorsman

**Affiliations:** 1 Department of Clinical Genetics, Amsterdam UMC, Location VUMC, De Boelelaan 1117, 1118, 1081 HV Amsterdam, The Netherlands; 2 Medpace, Cincinnati, OH 45227 USA; 3 Division of Oncology, Cincinnati Children’s Hospital Medical Center, Cincinnati, OH 45229 USA

**Keywords:** Cancer, Computational biology and bioinformatics

## Abstract

Head-and-neck squamous cell carcinomas (HNSCCs) are relatively common in patients with Fanconi anemia (FA), a hereditary chromosomal instability disorder. Standard chemo-radiation therapy is not tolerated in FA due to an overall somatic hypersensitivity to such treatment. The question is how to find a suitable alternative treatment. We used whole-exome and whole genome mRNA sequencing to identify major genomic and transcriptomic events associated with FA-HNSCC. CRISPR-engineered FA-knockout models were used to validate a number of top hits that were likely to be druggable. We identified deletion of 18q21.2 and amplification of 11q22.2 as prevailing copy-number alterations in FA HNSCCs, the latter of which was associated with strong overexpression of the cancer-related genes *YAP1, BIRC2, BIRC3* (at 11q22.1-2). We then found the drug AZD5582, a known small molecule inhibitor of BIRC2-3, to selectively kill FA tumor cells that overexpressed BIRC2-3. This occurred at drug concentrations that did not affect the viability of untransformed FA cells. Our data indicate that 11q22.2 amplifications are relatively common oncogenic events in FA-HNSCCs, as holds for non FA-HNSCC. Therefore, chemotherapeutic inhibition of overexpressed BIRC2-3 may provide the basis for an approach to develop a clinically realistic treatment of FA-HNSCCs that carry 11q22.2 amplifications.

## Introduction

Fanconi anemia (FA) is a rare hereditary disease characterized by bone marrow failure, congenital anomalies and predisposition to cancers, mainly leukemia and head-and-neck squamous cell carcinomas (HNSCCs)^1,2^. FA is caused by mutations in 1 of at least 22 genes collectively defined as the FA pathway that conducts the repair of DNA inter-strand crosslinks. Thus, at a cellular level, FA defects lead to hypersensitivity to DNA inter-strand cross linkers (ICLs), radial chromosomal breaks and cell death^1,2^.

HNSCCs are the most frequently occurring solid tumors in FA and they were originally diagnosed in 3–5% of all FA patients^3,4^. However, since successful bone marrow transplant procedures in the recent years have increased FA survival rates significantly, the frequency of HNSCCs in FA sharply increased and is expected to rise further. FA-HNSCCs are highly aggressive, which is reflected by a poor survival rate of approximately two years after diagnosis^4^. Due to the hypersensitivity of all somatic cells of FA patients to ICLs, this standard cancer treatment often causes severe and potentially fatal side effects in FA patients^4,5^. Radiation-based therapies are also used in treating FA-HNSCC. However, these approaches still offer limited therapeutic efficacy and can also lead to an array of complications such as mucositis, hematologic anomalies and dysphagia^5^. Consequently, in the FA community there is an urgent need for the development of safe alternative cancer therapeutics.

An essential theme in the development of efficient FA-HNSCC therapeutics, is the detection of cancer vulnerabilities that could be exploited to inhibit cancer cell growth with no significant negative effect on the normal FA cells and tissues. Cancer progression involves selection for somatic genomic alterations that enhance cell growth, collectively defined as cancer drivers^6^. Since normal FA cells already display fragility, oncogenesis in FA may select for compensatory mechanisms for survival. Hence, we hypothesized that a feasible strategy in the development of tailored therapeutics is to detect and target major genomic or transcriptomic alterations in FA-HNSCC that exclusively promote growth in FA-HNSCCs, while being nonessential in FA normal cells.

Detection of true oncogenic drivers can be complicated by the noise resulting from numerous passenger events that are characteristic of cancer. This is especially true for genomically unstable tumors, such as in FA. Integrative multi-level omics analysis can reduce data complexity and enhance target candidate selection. Moreover, the use of FA cancer cell lines combined with matched normal FA cell panels may allow to explore the functional consequences of the observed changes, and may also be exploited to test candidate drugs. The use of such an approach of matched tumor and normal cell panels is especially important for patient populations wherein normal cells are already genetically compromised.

The present study characterizes major genomic/transcriptomic components as well as copy- number-derived gene expression in FA-HNSCC. We aimed to investigate whether FA-HNSCC cell lines are characterized by HNSCC-general and/or FA-specific driver events that can be targeted. Herein, we identified 11q22.1-2 amplification as an important oncogenic event in the majority of FA-HNSCC cell lines. Moreover, functional analysis indicates that *BIRC2-3 a*re promising, tumor-cell specific druggable targets in FA-HNSCC cells with the 11q22.2 amplification.

## Results

### Somatic mutation analysis of FA-HNSCCs

#### High frequency somatic mutations in FA-HNSCC are limited to TP53

**Figure 1:**
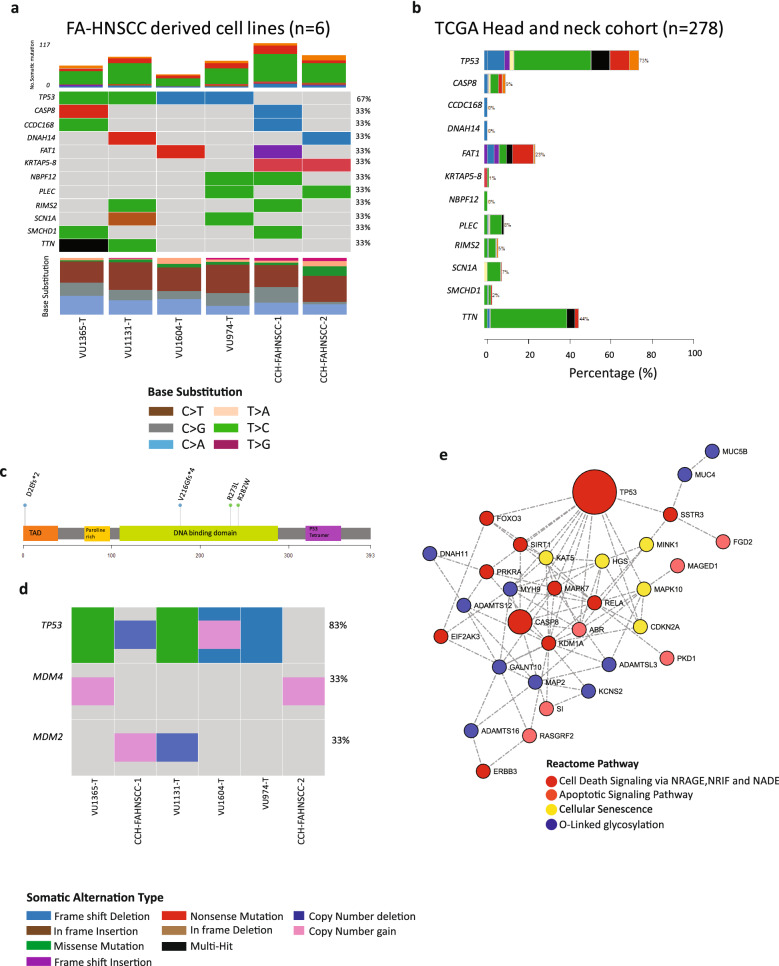
Somatic mutation analysis in FA-HNSCC. Frequently mutated genes in FA-HNSCC are limited to TP53. (**a**) Oncoplot for top frequently mutated genes in FA-HNSCC tumor cells (≥ 33%). Genes are shown in rows. Each column depicts mutations in each sample. Stacked bars on the top present number of mutations as well as mutation type per sample. Stacked columns on the bottom present nucleotide substitution frequencies. *TP53* is the only gene that is frequently mutated across FA-HNSCCs. (**b**) The status of FA-HNSCC frequently-mutated genes in TCGA, head- and-neck cancer cohort. (**c**) Oncoplot depicting the *TP53*-*MDM2/4* genomic state in FA-HNSCC. The two *TP53* non-mutated samples CCH-FAHNSCC-1 and CCH-FAHNSCC-2 indicate deep deletion of *TP53* and a gain of *MDM4* respectively. Consequently, deactivation of the *TP53* network appears to be the common denominator among FA-HNSCC derived cell lines. (**d**) Functional gene interaction network. Depicts major affected functional gene networks in FA-HNSCC. Interaction analysis by GeneMANIA. Pathway analysis performed by Reactome. Nodes represent genes. Edges represent physical/pathway interactions. Node sizes are adjusted based on the occurrence of the mutations in FA-HNSCC. Maximum node size; occurrence = 4. Minimum node size; occurrence = 1. Node colors adjusted based on associated pathways. Apoptotic/cellular senescence are the major mutated pathways in FA-HNSCCs. Apart from *TP53*, other pathway-associated genes are mutated in a cell line-specific fashion.

Somatic mutation analysis was based on whole-exome sequencing of a panel of six FA-HNSCC derived cell lines across three different genotypes FA-A (n = 4), FA-C (n = 1) and FA-L (n = 1) In total, 453 non-synonymous or high-impact somatic variants at population frequency < 0.01 were detected with an average of 71 variants per sample (Fig. 1a, Supplementary Excel File 1). Missense mutations were the most frequent variant type, followed by nonsense mutations. C > T was the most dominant base substitution. The ranking based on mutation frequency detected only 11 genes with a mutation frequency of 33% (Fig. 1a). *TP53* is the only gene mutated in the majority of FA-HNSCC derived cells with four out of six having missense or frameshift mutations This is in accord with the TCGA sporadic HNSCC cohort data (n = 278), where *TP53* was the most frequently mutated gene (Fig. 1b). A total of three out of four *TP53*-mutated samples harbored an allele frequency (AF) of one (Supplementary Table 1). All missense *TP53* mutations detected in FA-HNSCCs were previously reported by COSMIC in multiple cancers. A total of three out of four *TP53* mutations in FA-HNSCC cell lines reside in the *TP53* DNA-binding domain, while one mutation was located in the transactivation domain (Fig. 1c).

Subsequent somatic copy number analysis (SCNA) detected a deep deletion of *TP53* (Log_2_ Ratio <  − 1) as well as *MDM2* gain (Log_2_ Ratio > 0.3) in CCH-FAHNSCC-1 and a gain of *MDM4* in CCH-FAHNSCC-2 (Fig. 1d). Furthermore, VU1365-T, the only sample with a heterozygous *TP53* mutation, also bears a *MDM4* gain. Thus the *TP53* network was affected in all tumors, although via distinct mechanisms.

Functional gene–gene interaction analysis identified apoptosis and cellular-senescence as the most affected DNA-mutation-level pathways in FA-HNSCCs (Fig. 1e). With the exception of *TP53*, there was no significant selection for particular cell-death associated genes with different genes belonging to common pathways mutated in different cell lines. Overall, the mutation analysis points to apoptosis and cell death related networks in general and the abrogation of the *TP53* in particular as the most prevalent mutation-level somatic event in FA-HNSCC.

### Somatic copy number alteration (SCNA) analysis

#### FA-HNSCCs are associated with significant amplification of 11q22.2 and 18p deletions

**Figure 2:**
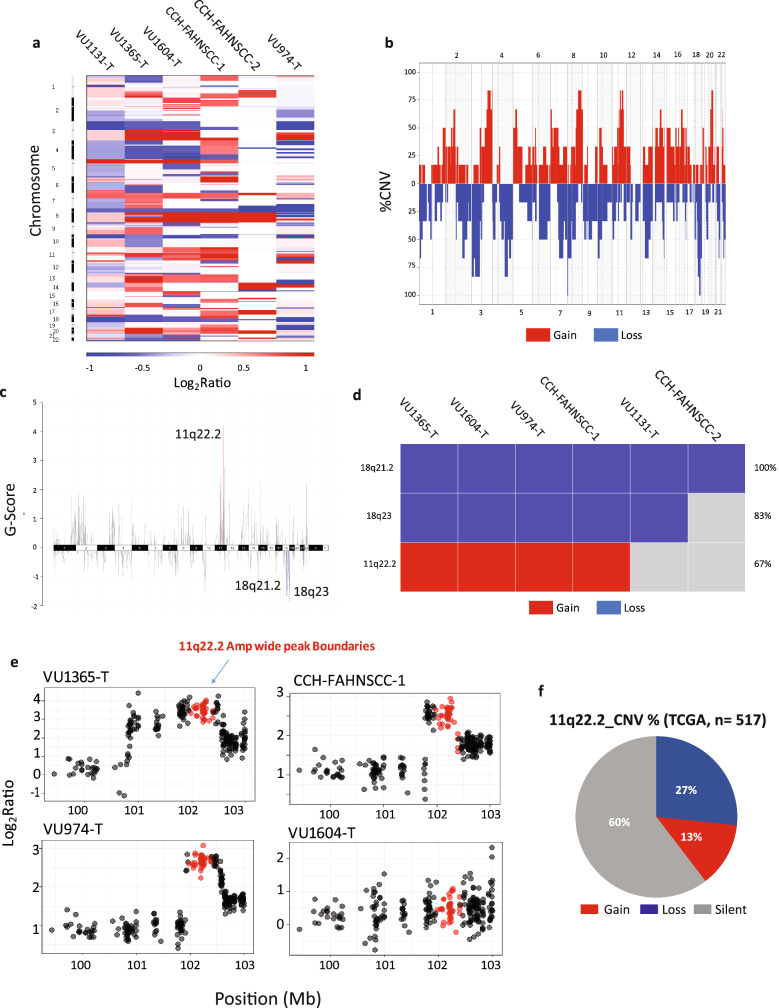
Somatic copy number alternation (SCNA) analysis in FA HNSCC cells. FA-HNSCC cell lines are characterized by significant amplifications of 11q22.2 and deletions of 18q21.2/q23. (**a**) Copy number heat map, representing somatic variations across samples. Columns represent samples. Rows represent chromosomes. FA-HNSCC cell lines show a high degree of aneuploidy with the exception of CCH-FAHNSCC-2. (**b**) Copy number frequency plot. The frequency of aneuploidy at each genomic position throughout FA-HNSCCs has been indicated. Y axis: Percentage of SCNA, X axis: chromosomes. Multiple chromosomes displayed high frequency of SCNA. (**c**) GISTIC genome plot for significance of SCNA in FA-HNSCCs across chromosomes. The q-Value cut-off was set at < 0.05. Y-axis: G-Score (Amplitude + Frequency), X-axis: Chromosomes. Positive G-Scores were associated with the significance of amplifications. Minus G-Scores were associated with the significance of deletions. The 11q22.2 amplifications and the 18q22.2/q23 deletions were the most significant SCNAs in FA-HNSCC derived cell models. (**d**) Oncoplot for GISTC significant SCNAs represents the frequency of each significant alternation. (**e**) Genomic copy number plot depicts the position and alternation pattern of q22.1-2 amplification wide peak boundaries on chromosome 11. Y-axis presents copy number Log_2_ Ratio, X-axis pinpoints genomic coordinates in Mb, 11q22.2 wide peaks are colored in red. In total 3 out of 4 FA-HNSCC samples harbored a focal amplification of 11q22.2 on the pre-existing gained background. VU1604 displayed a gain (**F**) Pie chart, representing the percentage of TCGA sporadic head-and-neck cancer cohort (n = 517) with 11 q22.2 copy number variation. Region 11q22.2 is amplified in 13% and it is deleted in 27% of sporadic samples. Copy number values as Log_2_ Ratio (Tumor/normal). Gain (Log_2_ Ratio > 0.3), Loss (Log_2_ Ratio < -0.3).

SCNA analysis was based on whole-exome sequencing reads obtained from 6 FA-HNSCCs and a pool of 5 FA normal and Cancer Associated Fibroblasts (CAFs) (Fig. 2, Supplementary Excel Files 2–4) The Log_2_ ratios larger than 0.3 and less than − 0.3 were considered as gains and losses respectively). The SCNA analysis detected massive and frequent copy number variations in all FA-HNSCC cell lines with the exception of CCH-FAHNSCC-2 (Fig. 2a). FA-HNSCC samples showed high occurrence of q arm amplifications of chromosomes 3, 8, 11, and 20, while chromosomes 3, 4, 18 and 21 displayed frequent deletions (Fig. 2b).

To detect potential cancer-driver regions, GISTIC analysis was performed to call significantly altered regional and focal SCNAs based on amplitude and frequency. GISTIC analysis at a confidence level of 0.75 and a q-Value cut-off of < 0.05 reported 11q22.2 amplifications (q-Value = 0.0025, frequency = 66%), 18q23 deletion (q-Value = 0.0315, frequency = 83%) and 18q21.2 deletions (q-Value = 0.0455, frequency = 100%) as the most significant copy number altered loci in FA-HNSCC cell lines (Fig. 2c,d, Supplementary Excel File 5).

The 11q22.2 amplification wide-peak boundaries range from chr11:101.9–102.4 (Mb), covering the extremity of 11q22.1 as well as the 11q22.2 cytoband (Fig. 2e). The region encompasses six genes; the hippo-pathway associated *YAP1, MMP7*, Cilia and Flagella associated *CFAP300* (*C11orf70*), the oncosis-associated and Porimin encoding *TMEM123* and the inhibitors of apoptosis family members *BIRC2-3*. The VU1365-T, VU974-T and CCH-FAHNSCC-1 cell lines showed extreme focal 11q22.1-2 amplifications (Log_2_ Ratio > 2). VU1604 harbors a single gain (Log_2_ Ratio > 0.3). Cross comparison with TCGA SNP array copy number data for 517 sporadic samples based on identical copy number gain and loss parameters, showed recurrent deletions and amplifications of 11q22.1-2 in 27% and 13% of sporadic HNSCCs respectively (Fig. 2f).

While the 18q23 deletion was confined to a relatively small genomic region, comprising two previously characterized genes, *ADNP2* and *PARD6G*, the 18q21.2 wide peak boundaries encompassed a large genomic region between chr18:40.6–76.84 (Mb) and constitutes 153 genes, including TGF-beta family genes *SMAD2, SMAD4* and *SMAD7* (Supplementary Fig. 1A).

### FA-HNSCCs transcriptomic analysis

#### FA-HNSCCs indicate significant transcriptomic differences when compared to SP-HNSCCs

**Figure 3:**
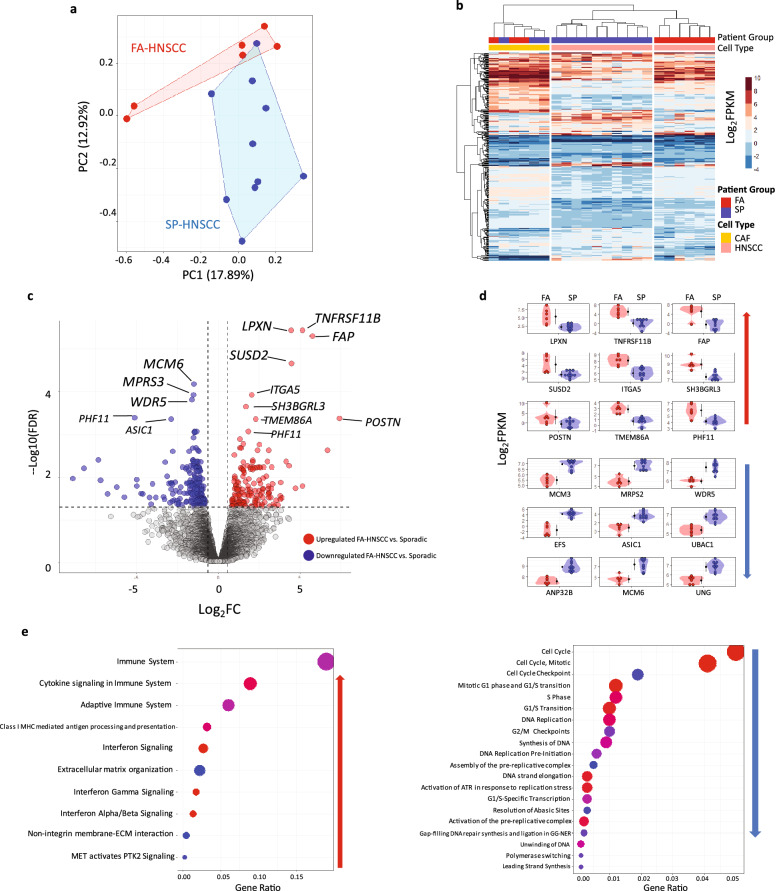
Transcriptomic analysis of FA/SP-HNSCC cell lines. FA-defects confer significant expression differences. (**a**) Principal component analysis (PCA) with Partition Around Medoids (PAM). Based on normalized expression values of all genes with at least minimum expression (CPM > 2). PAM clustering was set at two (k = 2). FA and SP samples were interrelated for the first PC of expression, and they only show a partial overlap according to the second PC. (**b**) Hierarchical clustering with heatmap for 363 differentially expressed genes (DEGs) between FA and SP HNSCCs (FDR < 0.05). Columns represent samples. Rows represent genes. The results reveal distinctions between FA and SP HNSCCs, but not between the cancer-associated FA and SP fibroblasts (CAFs). (**c**) Volcano plot for FA-HNSCC DEGs. (**d**) Violin-Dot plots for top FA-HNSCCs DEGs (FDR < 0.001). The top panel represents FA-HNSCC-upregulated genes. The bottom panel shows the top downregulated genes in FA-HNSCC cells. (**e**) Bubble plots for the significantly differentially expressed pathways in FA-HNSCC compared to SP. Pathway analysis was done with Reactome. Pathways were ranked based on gene ratio (Gene in list/Genes in pathway). FA-HNSCC cells were associated with upregulation of immune response/interferon-signaling associated pathways and downregulation of cell-cycle related pathways compared to SP-HNSCCs. Expression values as normalized Log_2_ FPKM, FA Upregulated (Log_2_ FC > 0, FDR < 0.05), FA downregulated (Log_2_ FC < 0, FDR < 0.05).

To further examine the functional consequences of these genomic changes as well as to characterize major transcriptomic features of FA-HNSCC, mRNA expression profiles of six FA, ten sporadic (SP) head and neck cancer cell lines as well as three FA and SP paired CAFs were obtained by genome-wide RNA-Seq. The relationships between normalized expressions Log_2_ FPKM of the samples were examined by Principal Component Analysis (PCA), overlapped with the Partition Around Medoid (PAM) (Fig. 3a). PAM was based on 2 cluster criteria (k = 2). While the FA and SP samples were interrelated based on the first Principal Component (PC), the FA samples tend to cluster together based on the second PC with only a partial overlap between SP and FA expression patterns, according to the PAM analysis. In order to identify genes responsible for the observed differences between FA and SP, differential expression analysis (DEA) was performed (Fig. 3b–d, Supplementary Excel File 6). In total, 363 differentially expressed genes (DEGS) were identified between FA-HNSCC versus SP-HNSCC (FDR < 0.05) with 150 upregulated (Log_2_ FC > 0, FDR < 0.05) and 213 downregulated genes (Log_2_ FC < 0, FDR < 0.05). Hierarchal clustering showed a clear separation between FA and SP tumors for the DEGS (Fig. 3b). In parallel, we also tested the behavior of these 363 DEGs in the patient-matched FA and non-FA CAFs. However, these genes did not show significant differences in expression between these two groups.

The cell-signaling tyrosine kinase associated *LPXN* and the TNF Receptor Superfamily Member 11b, *TNFRSF11B* were the top upregulated genes in FA-HNSCC. The DNA replication factor *MCM6*, the mitochondrial ribosomal factor *MRPS2* were the most significantly downregulated genes in FA-HNSCC compared to SP cells (Fig. 3c,d).

#### FA-HNSCC cells show upregulation of immune and interferon response associated genes compared to SP-HNSCCs

Functional annotation for genes upregulated in FA-HNSCCs (Log_2_ FC > 0, FDR < 0.05), using Reactome pathway analysis identified multiple overlapping pro-inflammatory related pathways such as those linked to the immune response, cytokine production and interferon signaling (Fig. 3e, Supplementary Fig. 2A–B). Several of these highly expressed genes have growth inhibitory and/or tumor suppressor functions (e.g. *XAF1*, *SP100*). Pathway analysis for genes downregulated in FA-HNSCCs (Log_2_ FC < 0, FDR < 0.05) revealed several overlapping pathways associated with cell cycle and replication.

#### Correction of the FA core-complex defect reduces the overall expression of immune response pathway associated genes

To examine the role of FA core-complex defect in the emergence of the FA-tumor specific differentially-expressed pathways, the global mRNA expression profiles, as obtained via genome-wide RNA sequencing, of the *FANCA*-deficient, FA-HNSCC cell line VU1365-T carrying an empty vector plasmid (VU1365 + EV) were compared to its *FANCA*-gene corrected counterpart VU1365 + FANCA (Supplementary Excel File 7). Differential expression analysis identified 4108 DEGs, with 2337 genes downregulated (Log_2_ FC < 0, FDR < 0.05) and 1771 upregulated (Log_2_ FC > 0, FDR < 0.05) in VU1365 + FANCA compared to its *FANCA* deficient counterpart. A Reactome Pathway analysis showed “interferon signaling” and the “immune response’s antigen presentation” among the most significantly downregulated pathways (FDR = 4.45 × 10^−14^) (Supplementary Fig. 3A). In line with this, 35 interferon-related genes showed significant reduction in expression in VU1365 + FANCA compared to its FANCA-deficient counterpart (Supplementary Fig. 3B–C). Overall the analysis of the isogenic-pair mRNA expression data supports a role for the FA core complex defect in the induction of inflammation associated interferon and immune response genes.

### SNCA-gene expression correlation analysis

#### Copy number changes strongly drive gene expression in FA-HNSCC

**Figure 4:**
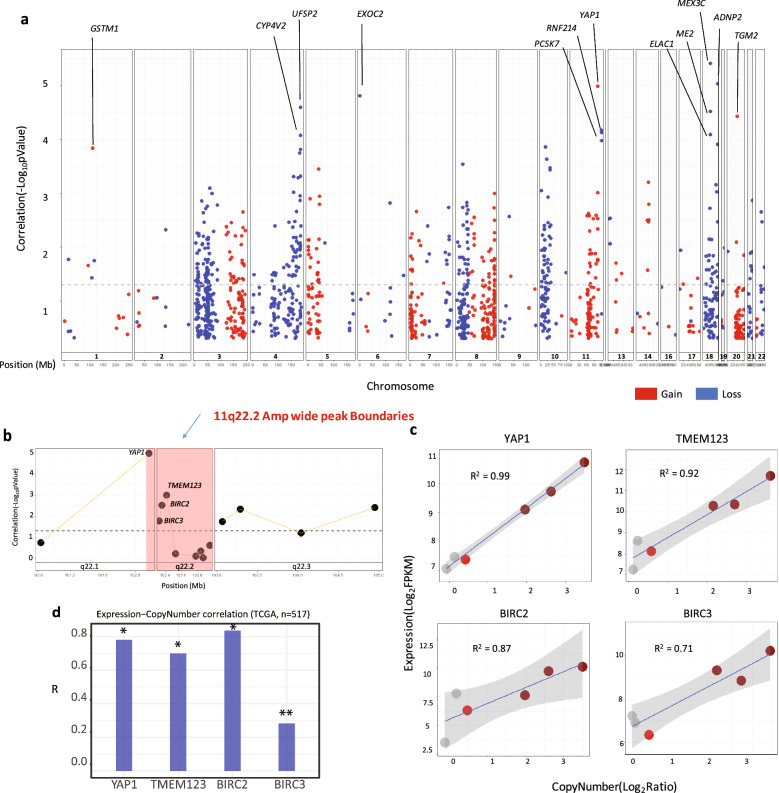
FA-HNSCC Copy number-expression correlation analysis. Elevated copy number correlates strongly with expression levels of genes within 11q22.1-2 amplification boundaries. (**a**) Genomic point plot depicts the significance of copy number-expressions in FA-HNSCC. Only genes with correlation (R) > 0 and average copy-number Log_2_ ratio > 0.3 or < 0.3 are shown. Y-axis represents correlations minus log_10_-transformed *p* Value. X-axis represents chromosomes and genomic coordinates in Mb. Significant correlations are above the dashed line (*p* Value < 0.05). *GSTM1*, *YAP1* and *TGM2* amplifications are strongly correlated with gene expression. (**b**) Genomic point plot for the significance of copy number-expression correlations of 11q22.1-2 genes. Y-axis represents correlations minus Log_10_-transformed *p* Value. X axis presents chromosomes and genomic coordinates in Mb. Correlations above the dashed line (*p* Value < 0.05) are significant, showing increased expression of *YAP1*, *BIRC2&3* as well as *TMEM123* related to copy number alterations. (**c**) Scatter plots for individual genes with significant copy number-expression correlations on 11q22.1-2 segment across FA-HNSCCs. Y-axis copy number (Log_2_ Ratio). X-axis expression (Log_2_ FPKM). Amplified samples (Log_2_ Ratio > 1). Gain (Log_2_ Ratio > 0.3). (**d**) Bar chart depicts the copy number- expression correlations (R) for *YAP1, TMEM123, BIRC2–BIRC3* in TCGA’s sporadic HNSCC cohort. *YAP1* and *BIRC2* show the strongest positive correlations. All correlations are significant. *q-Value < 10^–10^, **q-Value < 10^–5^.

To identify genes with copy-number associated expression changes, Pearson correlation analysis was performed based on the normalized copy number (Log_2_ ratios) and expression (Log_2_ FPKM) (Fig. 4, Supplementary Excel File 8). Correlations with R > 0 and *p* Value < 0.05 were considered as significant. Out of the tested 11,816 genes with minimum accepted overall mRNA expression (i.e. CPM > 2 in 33% of samples), 2139 genes had significant copy number-expression correlations with a total of 133 genes showing overall gain in copy number (Average Log_2_ ratio > 0.3) and 260 genes harboring overall loss (Average Log_2_ Ratio <  − 0.3). 1743 of the correlated genes were residing in regions with overall copy number value below or above the defined gain and loss thresholds. To detect most biologically meaningful SCNAs, applicable to the majority of FA-HNSCC cell lines, the analysis was focused on significant correlations (R > 0, *p* Value < 0.05), exhibiting average copy number defined as gain or loss throughout the FA-HNSCC cell lines (Fig. 4a).

Chromosomes 3, 4, 8, 10, 11 and 18 harbor the largest number of genes with copy-number driven expression. The cancer-related *GSTM1*, *YAP1* and *TGM2* genes showed most significant correlation with expression among the FA-HNSCC amplified genes. A cluster of genes on the 18q21.2 deletion region showed around 100% correlation with gene expression. These include *MEX3C, ADNP2, ME2*, *ELAC1*, and *UFSP2.* The *CYP4V2* on chromosome 4 and *EXOC2* on chromosome 6 were also among the top expression-copy number correlated genes, which showed overall deletion in FA-HNSCCs (Fig. 4a, Supplementary Fig. 1b).

#### The expression levels of YAP1, BIRC2-3, TMEM1232 on 11q22.1-2 are strongly amplification driven

The amplification of 11q22.2 was shown to be the most significant SNCA by GISTIC. The proto-oncogene *YAP1* on wide-peak boundaries of 11q22.1 showed the most significant copy number and expression correlation among the amplified genes in FA-HNSCC (R^2 = 0.99, *p* Value = 0.00001) (Fig. 4a–c). In addition, correlation analysis indicated a strong tendency for elevated expression for the oncosis-associated *TMEM123* gene (R^2 = 0.92, *p* Value = 0.0009), the inhibitor of apoptosis *BIRC2* gene (R^2 = 0.87, *p* Value = 0.002) and the *BIRC3* gene (R^2 = 0.71, *p* Value = 0.01). *MMP7* and *C11orf70* on 11q22.2 amplification did not show significant correlations with expression (Fig. 4b–c). The copy number derived expression of 11q22.1-2 amplification resident genes was also observed in a broader cancer context; the TCGA sporadic HNSCC copy number—expression correlation data also showed significant positive correlations, most strongly in *YAP1, BIRC2* and *TMEM123* (Fig. 4d).

We also determined whether these genes were differentially expressed between FA and sporadic HNSCC tumor cells. The differential expression analysis between FA and SP HNSCCs has not revealed a statistically significant difference in the expression of these genes (Supplementary Excel File 6).

#### FA-HNSCC samples with 11q22.2 amplification exhibit an increase protein expression of YAP1 and BIRC3

**Figure 5:**
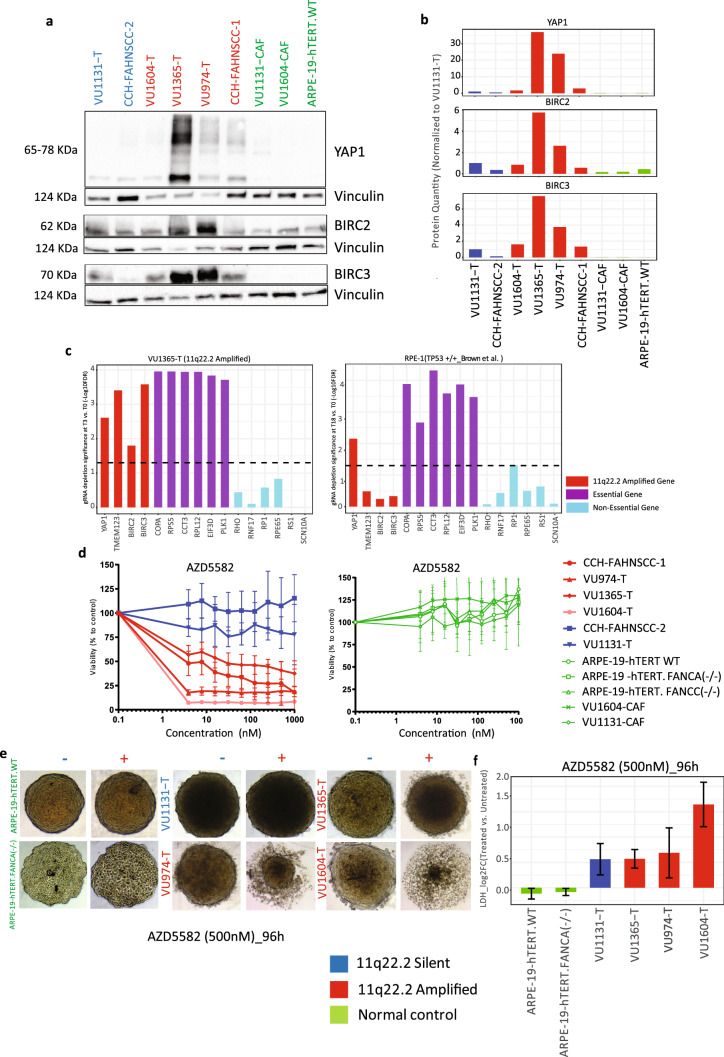
YAP1 and BIRC2-3 functional roles in FA-HNSCC cellular viability. The inhibition of BIRC2-3 selectively inhibits growth of FA-HNSCC cell lines with 11q22.2 amplification and no adverse effects on normal cells. (**a**) YAP1/BIRC2-3 western blots depict protein expression in FA-HNSCCs and a panel of normal controls. Vinculin is utilized as a loading control. YAP1, BIRC2 and BIRC3 proteins are most strongly expressed in the FA-HNSCC panel. (**b**) Bar charts representing YAP1, BIRC2 and BIRC3 protein levels in relation to vinculin and normalized to 11q22.2 silent VU1131-T. FA-HNSCC samples harboring 11q22.2 amplification show overall elevated expression of YAP1 and BIRC3 proteins, compared to 11q22 silent HNSCCs and a panel of normal immortalized cells. (**c**) Bar charts depicts the overall depletion significance (− Log_10_FDR) of gRNAs targeting 11q22.2 amplified genes and a panel of known essential and known non-essential genes in VU1365-T (left) and RPE-1 cells (Right). Depletions with FDR < 0.05 were considered significant. All gRNA targeting the selected 11q22.2-residing genes displayed significant sensitizing effects, in ranges comparable to guides targeting known essential genes. *YAP1* turned out to be the only 11q22.2 amplification gene with its gRNAs significantly depleted in both VU1365-T as well as the normal cell model, suggesting a more general vitality role for *YAP1.* (**d**) Dose response with the BIRC2/BIRC3 inhibitor AZD5582. FA-HNSCC cells are depicted on the left and untransformed cells on the right. AZD5582 treatment for 96 h resulted in significant reduction in viability in FA-HNSCC with 11q22.2 amplification compared to 11q22.2 silent tumors or normal cells. VU974-T and VU1604 exhibit the most dramatic response to the drug treatment and reach IC50 by 3.9 nM. VU1365-T was the most resistant 11q22.2 amplified cell line with AZD5582 and reached IC50 at 250 nM. None of the 11q22.2 silent or normal cells show an adverse negative response to AZD5582 in concentration ranges between 3.8–1000 nM. (**e**) AZD5582 treatment of a panel of FA-HNSCC and normal spheroid cultures. Treatment with AZD5582 (500 nM) for 96 h resulted in cell detachment or spheroid disintegration specifically in 11q22.1 amplified FA-HNSCC cells. (**f**) Lactate dehydrogenase (LDH) cytotoxicity assay. Y axis indicates the Log_2_-transformed fold changes in LDH level between AZD5582-treated and untreated samples.

Due to the significance of 11q22.2 amplifications, strong copy number-gene expression correlations and the known oncogenic properties of *YAP1*, *BIRC2* and *BIRC3*, we further tested their potential functional roles in sustaining cellular growth using available inhibitory compounds. Western blot analysis was performed to determine the presence of YAP1 and BIRC2-3 proteins in the 11q22.2 amplified samples compared to the 11q22.2 silent FA-HNSCC cells. To achieve this, the levels of the proteins of interest have been compared to the levels in the VU1131-T cell line, which does not show amplification. In parallel, the levels of the same proteins were also determined in matched normal cell lines; CAFs as well as the wild type retinal cell line model ARPE-19-hTERT.WT (Fig. 5a,b, Supplementary Fig. 4). Western blotting confirmed the expression of YAP1, BIRC2 and BIRC3 proteins, mostly pronounced in FA-HNSCC’s 11q22.2-amplified samples (Fig. 5a). The tested untransformed cells did not show a detectable level of YAP1 and BIRC2 protein expression. The protein quantification in relation to vinculin and normalized to 11q22.2 silent FA-HNSCC sample VU1131-T indicated higher protein expression of YAP1 and BIRC3 in all 11q22.2 amplified FA-HNSCCs compare to the 11q22.2 silent and normal cell lines (Fig. 5b). Protein levels of YAP1, BIRC2 and BIRC3 were most strongly elevated in VU1365-T and VU974-T cells, in line with the 11q22.2 copy number variation profile. Although the BIRC2 protein expression in VU1604-T and VU974-T were higher than the 11q22.2 silent CCH-FAHNSCC-2 and the panel of normal cells, they were comparable to that of VU1131-T. In summary, western blot analysis confirmed the overall elevation of YAP1, BIRC2 and BIRC3 expression in FA-HNSCC cell lines with 11q22.2 amplification.

#### YAP1, TMEM123 and BIRC2-3 are fitness genes in 11q22.2-amplified VU1365-T cells

For the VU1365 + EV cell line we had data from a genome-wide CRISPR screen using the TKOv3 library aimed at determining fitness genes at our disposal (unpublished data). The available gene lists were exploited to investigate how YAP1, TMEM123, and BIRC2-3 behaved in this analysis. The data indicated an overall significant depletion of gRNAs targeting *YAP1, TMEM123, BIRC2-3* in VU1365-T(T3) vs. VU1365-T (T0) (FDR_gRNAs depletion in T3 < 0.05) (Fig. 5c; raw sgRNA reads Supplementary Excel File 9). The significance of the depletions, particularly for guides targeting *YAP1, TMEM123* and *BIRC3* were comparable to those gRNAs targeting known essential genes and much stronger than guides targeting known human non-essential genes (Fig. 5c).

To examine whether, the negative selection for gRNAs targeting 11q22.2 residing genes was also observed in normal cells, the publicly available raw read counts from a pooled CRISPR screen for the normal cell model RPE-1 (TP53 ^+/+^) were analyzed^7^. From the four 11q22.2 amplification-region-residing genes only the *YAP1* gRNAs indicated a significant depletion. The depletion pattern for the essential and non-essential genes in RPE-1 also matched those gRNAs in VU1365-T (Fig. 5c). Overall the CRISPR data indicate that the 11q22.2 amplification residing genes *YAP1, TMEM123, BIRC2-3* are essential for VU1365-T tumor cells vitality.

#### Chemical targeting of BIRC2-3 selectively inhibits viability in the 11q22.2 amplified FA-HNSCCs but not in FA normal cells

To assess the consequence of YAP1 or BIRC2-3 inhibition on the viability of FA-HNSCCs with 11q22.2 amplifications, dose–response assays using AZD5582, a potent inhibitor of *BIRC2-4,* and CA3, an inhibitor of the YAP1-TEAD interaction were performed (Fig. 5d, Supplementary Fig. 5A). According to our expression-profiling results, *BIRC4*, nevertheless, did not show any detectable expression in the HNSCC cell lines. Therefore, an AZD5582 inhibitory effect is expected likely to be mediated exclusively via BIRC2 and BIRC3.

To determine whether a compound has the desirable effect in the context of FA, i.e. killing FA tumor cells and relatively sparing the normal cells, we analyzed both the tumor cells, the CAFs and the RPE cells.

Accordingly, to assess the differential drugs effect on normal cell viability alongside, a set of three ARPE-19-hTERT cell lines in WT, FANCA.KO, FANCC.KO genetic backgrounds, as well as the FA-HNSCC- matched normal cell lines, VU1131-CAF and VU1604-CAF, were included in the drug dose–response assays. The 11q22.2 silent FA-HNSCC cell lines were also included to evaluate the differential effect of the drugs based on the absence/presence of 11q22.2 amplification in tumor cells.

Addition of AZD5582 resulted in a significant reduction in viability in FA-HNSCC cell lines with 11q22.2 amplification compared to 11q22.2 silent FA-HNSCCs and normal cells (Fig. 5d). VU974-T and VU1604 exhibited the most extreme responses to the drug and reached the IC50 at 3.9 nM. VU1365-T was the most resistant 11q22.2-amplified cell line with an IC50 of 250 nM. None of the 11q22.2-silent or normal FA cells showed an adverse negative response to AZD5582 at the tested concentration range of 3.8-1000 nM.

11q22.2 amplified FA-HNSCC samples displayed a lower IC50 compare to the 11q22.2 silent and ARPE-19 samples for CA3 (350–500 vs. 750 nM). However, both the VU1604-T cancer cell line and its matched untransformed VU1604-CAF cells had similar IC50 values (Supplementary Fig. 5). Overall, the CA3 dose–response results were consistent with the CRISPR knock-out data, which suggested a negative effect of *YAP1* gRNA-induced knockout on cell growth in both 11q22.2 amplified VU1365-T as well as the normal ARPE-19 cell model.

To define the efficacy of AZD5582 under conditions that resemble tumor topology more closely, spheroid cell cultures were established from a panel of transformed and untransformed FA cells. These included 11q22.2 silent FA-HNSCC, VU1131-T, the 11q22.2 gained/amplified FA-HNSCCs, VU1604-T, VU974-T and VU1365-T, as well as the non-tumor 11q22.2 silent ARPE-19-hTERT WT and *FANCA.KO* (Fig. 5e)*.* The cell lines CCH-FAHNSCC-1 and CCH-FAHNSCC-2 were not analyzed, because spheroid cultures could not be established. The AZD5582 treatment (500 nM) for 96 h resulted in spheroid disintegration, mostly observed in VU1604-T, followed by VU974-T. The VU1365-T spheroids were less negatively affected by treatment, with mild yet visible cell detachment (Fig. 5e). The untransformed spheroid cultures as well as VU1131-T did not exhibit visible changes upon AZD5582 treatment. To measure the cytotoxic effect of AZD5582 on spheroid culture vitality, a lactate dehydrogenase (LDH) cytotoxicity assay was also performed (Fig. 5f). AZD5582 increased the LDH in the medium in all 11q22.2 amplified FA-HNSCC cell lines with highest elevations in VU1604 (Log_2_ FC_*Treated vs. Untreated*_: 1.6) and VU974-T (Log_2_ FC_*Treated vs. Untreated*_: 0.68). The VU1131-T also showed an increase in LDH levels (Log_2_ FC_*Treated vs. Untreated:*_ 0.56). None of the normal ARPE-19 cell models regardless of FA status showed increased LDH levels between the treated and untreated counterpart (Fig. 5f).

## Discussion

This study searches for targetable vulnerabilities in FA-HNSCC by defining major genomic and transcriptomic events in a unique panel of FA-HNSCC derived cell lines compared to normal immortalized FA cells. In contrast to previous hypotheses, we did not detect a ubiquitous targetable FA-cancer vulnerability. However, genomic analysis, identified mutations of *TP53* as the main common somatic variant, and the TP53 pathway seem to be disrupted in all tested FA-HNSCC cell models. The FA tumor cells may still suffer from persistent DNA damage, as suggested by the upregulation of pro-inflammatory immune/interferon-response genes. Amplification of 11q22.2 and deletions of 18q21.2/18q23 turned out to be the most significant somatic structural alternations in the FA-HNSCC cell lines. Copy number and expression analysis revealed strong correlations for a subset of the amplified cancer-related genes, *YAP1, BIRC2-3,* on 11q22. Chemical inhibition of *BIRC2-3* resulted in a significant decline in FA-cancer-cell viability in samples with 11q22.2 amplification, and importantly no adverse negative effect on FA normal cells. Consequently, we propose that *BIRC2-3* is an important oncogenic driver and a potentially safe and effective therapeutic target in a subset of FA-HNSCC patients with 11q22.2 amplification.

The mutational landscape of FA-HNSCC did not exhibit either many commonly occurring mutations or non-canonical FA-specific variants. Somatic mutation analysis pointed to *TP53* as the only gene frequently mutated in the majority of FA-HNSCC derived cell lines. This is in line with mutation data from the TCGA head-and-neck cancer cohort as well as the major whole-genome studies that detected *TP53* mutation in 75%-85% of HNSCC cases^8,9^. Our results are also consistent with recent studies, that also reported *TP53* mutations in FA-HNSCC-derived cell-line models^10,11^. A total of four out of six FA-HNSCC cell lines, harbored *TP53* mutations with 3 residing in the DNA binding domain (DBD) and one in the transactivation domain 1 (TAD). DNA-contact mutations of *TP53* are the most common mutations in *TP53*, which emphasize the importance for the transcriptional activity of *TP53*^12^. The TP53 TAD domain is also critical for the activation of the *TP53* DNA-damage response^13^. Furthermore, FA-HNSCC lines without *TP53* mutations either displayed deletion of *TP53* or copy number gain of *MDM4* which is also associated with TP53 repression. Consequently, the data indicate that the deactivation of *TP53* is a pivotal step in FA-HNSCC tumorigenesis. *TP53* is the central inducer of cellular cell cycle check point arrest, cellular senescence and apoptosis in response to stressors such as DNA damage and hypoxia^14^. We argue that deactivation of *TP53* is a primal trigger for FA-HNSCCs tumorigenesis by promoting the initial survival of damaged FA cells.

It has been noted that the deactivation of *TP53* does not relieve FA cells from cytotoxic and genotoxic stress resulting from sustained DNA damage. At the cellular level, FA tumor cells remain hypersensitive to ICLs. At the molecular level, the FA-HNSCC expression network may, nevertheless, be altered by this hypersensitivity invoked by FA-pathway deficiency. Interestingly, characteristic expression patterns of FA-HNSCC cells do not reflect pro-proliferative signatures, but rather compromised vitality. Compared to the sporadic tumor cells, FA-HNSCC display a systemic upregulation of genes and pathways that are involved in pro-inflammatory response such as cytokine production and immune system/interferon signaling. The upregulation of immune responses and IFN-stimulated gene expression may be linked to the DNA damage susceptibility in FA-HNSCCs. Several studies have shown that cytosolic DNA fragments, generated by DNA damage, can trigger an immune response and induction of IFN stimulated genes^15–17^. In line with these findings, expression data for FANCA-deficient FA-HNSCC cell line, VU1365-T, and its isogenic *FANCA*-corrected counterpart show that *FANCA* correction results in a significant downregulation of interferon and immune response genes. This may suggest that FA loss of function induces pro-inflammatory responses due to increased, persistent susceptibility to DNA damage. The elevation of pro-inflammatory cytokines and interferon response genes can trigger senescence, apoptosis and immunogenic cell death in cancer cells^18–21^. Consequently, tumorigenesis in FA may require further genetic alterations beyond deactivation of *TP53* to boost survival and cancer progression. The oncogenic selective pressure in FA-HNSCC might thus be influenced by DNA-damage-induced-cellular stress.

Our integrated genomic/transcriptomic analyses pointed to the amplification of 11q22.1-q22 and deletion of 18q22.1 as important oncogenic alteration in FA-HNSCC. Both variants were the most significant genomic changes in the FA-HNSCC cell line panel with respect to amplitude/frequency and impact on gene expression. While 18q22.1 event involves the loss of *SMAD4*, a potent tumor suppressor and modulator of TGF-Beta signaling pathway^22^, 11q21.2 amplification strongly elevates the expression of the potent proto-oncogene *YAP1* and inhibitors of apoptosis *BIRC2-3*.

The functional properties and overall high prevalence of 11q22.2 amplifications in FA head and neck cancers make this region a potential oncogenic target in FA-HNSCC. *YAP1* is a transcriptional co-activator, which induces the expression of growth-promoting genes and suppresses apoptosis through its interaction with the TEAD-family of transcription factors^23–25^. *BIRC2* and *BIRC3* are members of the inhibitor of apoptosis (IAP) family of proteins. They play various roles in anti-apoptotic, tumor necrosis factor (TNF) mediated and canonical/non-canonical NFkB signaling^26,27^. Moreover, frequent amplification of the 11q22.1 region had already been reported in TCGA and other HNSCC genomic characterization studies^13,28^. Together these findings further emphasize the potential oncogenic relevance and opportunities for clinical targeting of 11q11.1-2 amplification in HNSCC in general and FA-HNSCC in particular.

From a tumorigenic perspective, both *YAP1* and *BIRC2-3* qualified as potential candidate oncogenes in FA-HNSCC. However, the genetic pooled CRISPR screen data as well as the drug-response assays did not provide conclusive evidence for specific oncogenic roles of *YAP1* or its therapeutic value to FA-HNSCC. *YAP1* turned out to be a possible fitness gene in both 11q22.2 amplified VU1365-T as well as the normal cell line model RPE-1 (TP53 ^+/+^). Consistent with the CRISPR KO results, the inhibition of the *YAP1* transcriptional co-activating function by the specific compound CA3 resulted in cell death in 11q22.1 amplified/silent cancer cell lines and normal controls as well. The 11q22.1 amplified samples showed, nevertheless, a one to two-fold lower IC50 compared to normal cells. This narrow therapeutic window may clearly form an obstacle to clinical applicability of CA3 but nonetheless provides proof of concept for our approach to therapeutics. These results may also suggest general non-oncogenic functions of *YAP1* in cellular viability. However, we cannot rule out the possibility of a cytotoxic side effect of CA3 treatment unrelated to the *YAP1-TEAD* interaction inhibition as the cause of the observed drug response outcome. Therefore, determining the exact role of *YAP1* in FA-HNSCC may require additional analysis through further genetic targeting of *YAP1*, further analysis of drug response using other available inhibitors, and in vivo studies.

In the case of BIRC2-3, the drug-response assays and the CRISPR KO data confirmed *BIRC2-3* as a potent fitness gene in 11q22.1-2 amplified FA-HNSCC cell lines. It was shown that BIRC2–BIRC3 inhibitors can be considered as potential candidate therapeutics in FA tumor cells harboring 11q22.2 amplification. Pooled CRISPR-screen depletion analysis showed *BIRC2-3* to be fitness genes in VU1365-T, but not in the normal cell model RPE-1 (TP53 ^+/+^). Consistent with these findings, the *BIRC2,3,4* inhibitor AZD5582 significantly reduced vitality and/or caused cell death selectively in 11q21.2 amplified FA-HNSCC cells, with no negative effect on the viability of normal cells or FA-HNSCC. In this context it is important to note that *BIRC4* did not show a measurable gene expression in the HNSCC cell lines. This suggests that the decline in vitality observed upon AZD5582 treatment in 11q22.2 amplified FA-HNSCC cells specifically resulted via BIRC2 and BIRC3 inhibition.

Together these data suggest a crucial tumor-context-specific oncogenic role of *BIRC2-3* amplification in HNSCC, likely through their multi-functional and complex anti-cell death properties. The potential therapeutic value of targeting *BIRC2-3* is also strengthened by the diverse array of tested and specific BIRC inhibitors, which are currently available., apart from AZD5582 which is being tested as a safe HIV therapeutic^26^. Other compounds include birinapant, xevinapant and tolinapant, and these are currently in phase 1 or 2 of clinical trials.

In conclusion, our results indicate that at least a subset of oncogenic events in FA-HNSCC arise from genomic copy number changes that elevate the expression of genes that potently suppress cell death signaling and promote vitality. The BIRC2-3 amplification is such an oncogenic event. Our analyses support the potential benefits of adopting a personalized medicine approach for tackling HNSCC in FA patients. We propose to assess the prevalence of 11q22.1 amplification in broader cohort of FA-HNSCC patients, and to evaluate the clinical possibilities of BIRC2-3 inhibitor-based therapy. This may facilitate the development of customized clinical pipelines in detecting and selectively targeting *BIRC2-3* in 11q22.2 amplified tumors within and beyond FA. Moreover, defining exact mechanisms whereby *BIRC2-3* contribute to oncogenesis in FA cells will be crucial for a deeper understanding of FA-HNSCC biology and treatment.

## Material and methods

### HNSCCs cell lines

Sporadic and Fanconi anemia cell lines were established via published procedures^29^. The main features of these established cell lines have been published before^11,30–32^, and are summarized in Table 1. All cell lines were cultured in DMEM (GIBCO, Cat# 11330057) supplemented with 10% Fetal Bovine Serum (FBS) (GIBCO Cat# 10270) and 1 mmol/L sodium pyruvate. Cell lines CCH-FAHNSCC-1 and CCH-FAHNSCC-2 had been established by culturing on fibroblast feeder cells, followed by adaptation to DMEM in the absence of feeders. All cell lines used were HPV-negative. The cell lines SCC11B, SCC14C and SCC22A were obtained from Dr. T. Carey (University of Michigan, Ann Arbor, MI). The cell line FADU-RE was obtained from ATCC. NeoTERT-immortalized FA patient-matched Cancer-Associated Fibroblasts (CAFs) and NeoTERT-skin Fibroblasts (Fs) were maintained on IMDM (GIBCO, Cat# 31980030) + 10% FCS + 2x Embryomax Nucleoside supplement (Merck, Cat# ES-008-D). Immortalized ARPE-19 hTERT retinal pigment epithelial cells were maintained on DMEM-F12 (GIBCO, Cat# 31331028) + 10% FCS with 15 µg/mL Hygromycin B (Roche, Cat# 10843555001). The immortalized *FANCA* and *FANCC* knockout (KO) ARPE-19 hTERT cells were generated by Neon electroporation of a Cas9: trRNA: crRNA ribonucleoprotein (RNP) complex (the procedure will be described in a manuscript in preparation). Stable, isogenic *FANCA* complementation group, VU1365-T tumour cell lines have been generated by electroporating patient-derived FANCA-deficient VU – SCC 1365T cells with pIRES-Neo EV (Empty Vector control) or pIRES-Neo-FANCA-FLAG (FLAG-tagged Wild Type human FANCA construct) using the Amaxa Nucleofector system and selection with 300 ug/mL G418.

**Table 1:** The phenotypic/genotypic information of FA and sporadic HNSCC samples.

	Sample name	Patient group	Tissue	Tissue of origin	FA Group	Immortalization	ICL resistance	Expression (RNA-Seq)	Mutation-Copy Number (WES)
Cancer cell lines	CCH-FA-HNSCC-1	FA	SCC	Tongue	FA-A	NA	No	✓	✓
	CCH-FA-HNSCC-2	FA	SCC	Tongue	FA-A	NA	No	✓	✓
	VU1131-T	FA	SCC	Floor of mouth	FA-C	NA	No	✓	✓
	VU1365-T	FA	SCC	Mouth mucosa	FA-A	NA	No	✓	✓
	VU1604-T	FA	SCC	Tongue	FA-L	NA	No	✓	✓
	VU974-T	FA	SCC	Tongue	FA-A	NA	No	✓	✓
	VU1365-T + EV	FA	SCC	Mouth mucosa	FA-A	NA	No	✓	X
	VU1365 + FANCA	FA	SCC	Mouth mucosa	FA-A	NA	No	✓	X
	FADU-RE	SP	SCC	Hypopharynx	NA	NA	No	✓	X
	SCC11B	SP	SCC	Larynx	NA	NA	Yes	✓	X
	SCC14C	SP	SCC	Floor of mouth	NA	NA	Yes	✓	X
	SCC22A	SP	SCC	Hypopharynx	NA	NA	Yes	✓	X
	SCC9917	SP	SCC	Oral cavity	NA	NA	Yes	✓	X
	UPCI-154	SP	SCC	Tongue	NA	NA	No	✓	X
	VU041-T-RE	SP	SCC	Floor of mouth	NA	NA	No	✓	X
	VU40-T	SP	SCC	Tongue	NA	NA	Yes	✓	X
	VU120-T	SP	SCC	Tongue	NA	NA	Yes	✓	X
	VU94-T	SP	SCC	Tongue	NA	NA	Yes	✓	X
Untransforme cell lines	VU1131-CAF	FA	CAF	Stroma	FA-C	Neo-TERT	No	✓	✓
	VU1365-CAF	FA	CAF	Stroma	FA-A	Neo-TERT	No	✓	✓
	VU1604-CAF	FA	CAF	Stroma	FA-L	Neo-TERT	No	✓	✓
	VU1365-F	FA	F	Stroma	FA-A	Neo-TERT	No	X	✓
	VU1604-F	FA	F	Stroma	FA-l	Neo-TERT	No	X	✓
	VU120-T-F	SP	CAF	Stroma	NA	Neo-TERT	Yes	✓	X
	VU40-T-F	SP	CAF	Stroma	NA	Neo-TERT	Yes	✓	X
	VU94-T-F	SP	CAF	Stroma	NA	Neo-TERT	Yes	✓	X
	ARPE-19-hTERT.WT	NA	RPE	Retina	NA	h-TERT	Yes	X	X
	ARPE-19-hTERT.FANCA(−/−)	NA	RPE	Retina	FA-A (C-EK)	h-TERT	No	X	X
	ARPE-19-hTERT.FANCC(−/−)	NA	RPE	Retina	FA-C(C-EK)	h-TERT	No	X	X

All FA/HNSCCs are characterized by lack of FA-D2 monoubiquitation, hypersensitivity to ICLs and they carry pathologic mutations in FA genes. Three SP/HNSCCs exhibit sensitivity to ICLs.

*FA* fanconi anemia, *SP* sporadic, *SCC* squamous cell carcinoma, +*EV* empty vector plasmid transfected, +*FANCA* transfected with proficient *FANCA* plasmid, *CAF* cancer associated fibroblast, *F* fibroblast, *RPE* retinal pigment epithelial, *C-EK* CRISPR-engineered knockout, *NA* not applicable.

### Whole-genome RNA sequencing. mRNA extraction and library preparation

Cells were harvested during the exponential growth phase and approximately 10^6^ cells were used to extract RNA, using the High Pure RNA isolation kit (Roche, Cat# 11828665001). The TruSeq stranded mRNA sample preparation kit was used in preparing RNA-Seq library (Illumina Cat# 20020594).

### Sequencing and reads preprocessing

Six samples were pooled per lane and sequenced on an Illumina Hiseq2000 using the 100 bp paired-end protocol. The trimming of read ends and Illumina-adapter clipping were done with Trimmomatic v.0.36^33^. Reads were mapped to hg19 by STAR^34^, according to the UCSC gene human annotation table (https://genome.ucsc.edu/cgi-bin/hgTables). The SAM files were converted to the binary BAM, sorted and indexed with SAMtools ^35^. Subread was used in counting reads^36^.

### RNA sequence data analysis

Read counts normalization and differential expression analysis were done with edgeR v.3.30^37^. Only genes indicating at least 2 counts per million (CPM) in at least 33% of samples were included in the analysis. Data were normalized for sample-specific effects by the trimmed mean of M-values (TMM). This was followed by dispersion estimation and calling differentially expressed genes, using general linear model (GLM). False Discovery rate (FDR) < 0.05 hits were considered significant. Pathway analysis was done by web-based pathway analysis tool Reactome^38^. All graphs and plots were made using R/ggplot2.

### Whole-Exome sequencing (WES). Genomic DNA extraction and library preparation

Genomic DNA from six FA-HNSCC tumor cell lines, three paired cancer-associated fibroblast lines (CAFs) and two paired primary fibroblast lines were extracted with the QIAamp DNA mini kit, using the cultured cells protocol (Qiagen, Cat# 51106). DNA quantity was measured with Qubit 3.0 Fluorometer (Life technologies, Bleiswijk, The Netherlands). The DNA quality was measured by NanoDrop, 2000 (Thermo-Fisher Scientific, Massachusetts, United States). A secondary quality control was performed by DNA gel electrophoresis. The SureSelect human all exon V7 kit (Agilent, Cat# 5991-9039) was used in target enrichment and preparing exome library preparation, according to the protocol of the manufacturer (Agilent Technologies, Santa Clara, United States).

### Sequencing and reads processing

DNA pools were sequenced with 100 bp pair end reads at 30 GB per sample on Novaseq 6000, Illumina (Illumina, San Diego, United States). Sequence adapter clipping and quality trimming was carried out using Trimmomatic v.0.36^33^ Reads were mapped to hg19 with Burrows-Wheeler Aligner (BWA)^39^. BAM files were indexed and sorted by SAMtools^35^. Duplicate reads were marked with Picard.

### Somatic mutation analysis

Two parallel GATK best-practice pipelines were used in calling variants. The GATK4, variant caller MuTect2 was used in calling tumor specific somatic mutations^40^. A panel of pooled FA normal fibroblast/CAFs were used as control in calling somatic variants. Three out of six FA-HNSCC samples had their matched FA normal cells included in the panel of normal. All normal controls were sequenced with identical sequencing procedure and chemistry. Only variants marked as “PASS” were considered for further analysis. As many of the somatic variants passed by MuTect were not of high quality and may have been called due to sequencing errors, GATK standard variant caller Haplotypcaller was used in parallel to call variants and assess quality^40^. Based on GATK short variant hard filter criteria (https://gatk.broadinstitute.org/hc/en-us/articles/360035890471-Hard-filtering-germline-short-variants), SNPs matching any of the following parameters were flagged; QualByDepth (QD) < 2.0 | FisherStrand (FS) > 60.0 | RMSMappingQuality (MQ) < 50.0 | MappingQualityRankSumTest (MQRankSum) < −  12.5 | ReadPosRankSumTest (ReadPosRankSum) < −  8.0. Only variants passed by both variant callers were considered reliable. Variants were annotated by ANNOVAR^41^. Mutations were characterized based on population frequency by Exome Aggregation Consortium 03 (EXac03), common polymorphisms by the database of SNP (dbSNP), pathogenicity prediction by SIFT/Phred and clinical and cancer relevance by ClinVar and COSMIC respectively. While variants detected in FA-HNSCC cell lines with their matched normal controls generally showed very low or non-existing population frequency, the variants in samples without matched normal controls were further filtered based on EXac (frequency < 0.01) and dbSNP to cross out common polymorphisms. Moreover, variants reported as “Neutral” by SIFT/Phred database were excluded from the analysis. Mutations were summarized and visualized by R/MAFTOOLS v.4.0^42^. For functional network analysis, the “pathway” interactions between non-synonymous/high-impact variants were obtained initially using the gene–gene interaction database GeneMANIA (https://genemania.org/)^43^. Genes with known pathway interactions were analyzed for functional enrichment by Reactome (https://reactome.org/). Networks were built and visualized by Cytoscape v.3.4.0^44^.

### Somatic copy number analysis

Somatic copy number analysis based on whole-exome sequence reads was performed using the Python-based package CNVkit v.0.9.5^45^ The pool of patient CAFs and paired fibroblasts were used as control. Similar to Somatic mutation analysis, the panel of normal included the 3 patient matched cell lines. Genes with Log_2_ Ratio (Sample/Control) > 0.3 were considered as gain. Genes with Log_2_ Ratio (Sample/Control) < −  0.3 were considered as loss. The Genomic Identification of Significant Targets In Cancer 2 (GISTIC2 v.2.0.23) was utilized to identify significant copy number alterations based on frequency and amplitude^46^. GISTIC analysis was based on the following parameters; Confidence level = 0.75, gain = Log_2_ Ratio > 0.3, loss = Log_2_ Ratio <  − 0.3 and q-Value < 0.05^46^. R/MAFTOOLS v.4.0 was used in plotting GISTIC frequencies and scores. The GISTIC heatmap function was used to plot copy number heatmap. All the other copy number plots were made by R/ggplot2.

### Expression-copy number correlation analysis

Pearson Correlation analysis between matched normalized gene expression (log_2_ FPKM) and copy number Log_2_ Ratio (tumor/normal) was performed using R package MVisAGe v.0.2.1^47^. Correlations (R) > 0 with *p* Values < 0.05 were considered significant. R/ggplot2 was used in plotting correlations.

### Acquirement and processing of the sporadic HNSCC public genome data

TCGA somatic mutation annotation files (Mutation Packager_Calls, Sample # 279), SNP6 copy number segmentation file (genome_wide_snp_6-segmented_scna_minus_germline_cnv_hg19, Sample # 517) and copy number-mRNAseq expression correlation results were downloaded from the Broad GDAC Firehose Head and neck carcinoma data repository (http://firebrowse.org/?cohort=HNSC&download_dialog=true). Mutation annotation files were visualized and summarized with R/MAFTOOLS v.4.0^42^. The gene-level copy number estimates (Log_2_ Ratios) as well as significant copy number changes were obtained by GISTIC (Confidence level = 0.75, gain = Log_2_ Ratio > 0.3 loss = Log_2_ Ratio < − 0.3 and q-Value < 0.05)^46^.

### Pooled CRISPR screen and analysis

The whole-genome CRISPR screen for VU1365-T has been performed essentially, as previously described with a few modifications^48^. In brief, three populations of VU1365-T cells were transduced with a TKOV3 library at an infection rate of ~ 0.2 . Upon 24 h post-infection, the transduced cells were selected by addition of puromycin (5 µg ML^−1^ ). Subsequently, cells were collected before induction of Cas9 (T0) and after 8 and 12 population doublings (T2 and T3). Subsequently, DNA was isolated and sequenced. Obtained DNA sequences were mapped to their corresponding single guide RNAs (sgRNAs). Guides with no mismatch were considered for further analysis. Raw counts were scaled by total number of reads (i.e. CPM) and sgRNA and gene-level statistical testing between T3/T2 vs. T0 were performed based on Beta-binomial modeling, implemented in the R/CB2 package^49^. The sgRNA raw read counts for RPE-1 (*TP53* ^+/+^ pooled CRISPR screen were downloaded from the GEO repository (https://www.ncbi.nlm.nih.gov/geo/query/acc.cgi?acc=GSE128210)^7^. The list of reference essential and non-essential genes were obtained from the GitHub repository (https://github.com/hart-lab/bagel). Depletions and enrichment with FDRs < 0.05 were considered significant.

### Western blot analysis

The cell extracts from the six FA-HNSCC cell lines, two matched cancer associated fibroblast lines VU1604-CAF and VU1131-CAF, as well as the wild type (WT) ARPE-19 hTERT line were prepared by collecting and washing approximately 3–4 million cells in ice-cold PBS and collecting in RIPA Lysis and Extraction buffer (Thermo-Fisher Scientific Cat# 89900). Proteins were denatured in 1 times LDS sample buffer (NuPAGE, Cat# NP0007), by heating to 70 °C for 10 min. A total of 50 µg protein extracts from tumor and normal samples were separated by 8–16% gel (BioRad, Cat# 456-1104) and transferred onto activated PVDF membranes (Millipore, Immobilon-P, #IPVH00010 and ISEQ00010) with Towbin buffer (25 mM Tris, 192 mM Glycine, 10% Methanol) in BioRad TransBlot tank transfer systems. The Precision Plus Protein Dual Color Standards (Bio-Rad, # 161-0374) were used as molecular weight markers. Membranes were blocked in 5% milk or BSA in TBS-T (20 mM Tris–HCl, 150 mM NaCl, 0.1% w/v Tween20). The membranes were subsequently incubated overnight with YAP1 (Cell Signaling Technologies Cat# 4912)*,* BIRC2 & BIRC3 (IAP family antibody sampler kit, Cell Signaling Technologies Cat# 9970), and Vinculin (Santa Cruz, Cat# 25336) antibodies respectively. All primary antibodies were used at 1:1000 dilutions. Subsequently blots were washed 3 times and treated with secondary anti-rabbit (Dako, Cat# P044901-2) and anti-mouse antibodies (Dako, Cat# P044701-2). The secondary antibodies were used at 1:5000 dilutions. Blots were washed 3x in TBS-T, developed by chemiluminescent imaging with ECL prime western blotting detection reagent (ECL, Amersham, Cat# RPN2236) on a BioRad system. Blot visualization and protein quantification was performed by Image Lab (Biorad Hercules, California, United States).

### Drug inhibition response assay. 2D setup

The YAP1-TEAD interaction inhibitor CA3 (Selleckchem, Cat# S8661) and BIRC2-4 inhibitor AZD5582 (Selleckchem Cat# S7362) were used in the drug dose studies. The 6 FA-HNSCC tumor cell lines and their matched normal cell lines, VU1131-CAF and VU1604-CAF and non-tumor ARPE-19-hTERT wild type (WT), *FANCA*.KO, *FANCC*.KO cell lines, were included in the drug dose response assays. A total of 4000 cells from tumor cell lines and 5000 cells from normal cell lines were seeded in 96 well plates and incubated overnight. Cells were then treated with increasing concentrations of compound (1–1000 nM). After a total duration of 96 h the Cell-Titer blue assay was performed to assess viability (Promega, Cat# G8080) according to the manufacturer’s instructions. Each experiment was done in triplicate. Experiments were repeated in 3 independent settings. *3D setup* A total of 5000 cells were seeded in ultra-low attachment plates (Croning, Cat# CLS3474) in DMEM/F12 medium B27, EGF (5 ng/mL) and bFGF (5 ng/mL). The day after seeding, cells were treated with AZD5582 (500 nM). After 96 h, the effect of the drugs on viability were assessed by cell LDH-Glo Cytotoxicity Assay, according to the manufacturer’s instructions (Promega, Cat# J2380).

### Ethics approval and consent to participate

All FA patient-derived cell lines used in this study have been previously published.

## Supplementary Information

Supplementary Information 1.

Supplementary Information 2.

## Data Availability

*Mutation data* The FA-HNSCC cell lines’ somatic variant call file (VCF) is available in Supplementary File 1. *Copy number variation data* The FA-HNSCC cell lines’ somatic structural variant call file (SVCF) is available in Supplementary File 2. The FA-HNSCC cell lines’ copy number segmentation data are available in Supplementary File 3. The FA-HNSCC cell lines’ gene-level copy number values are available in Supplementary File 4. *Gene Expression data* RNA-Seq data has been deposited in the ArrayExpress database at EMBL-EBI (www.ebi.ac.uk/arrayexpress) under accession number E-MTAB-11167. Other data formats will be provided upon request.
