# Publisher Correction: *BIRC2–BIRC3* amplification: a potentially druggable feature of a subset of head and neck cancers in patients with Fanconi anemia

**DOI:** 10.1038/s41598-022-09471-8

**Published:** 2022-04-06

**Authors:** Khashayar Roohollahi, Yvonne de Jong, Govind Pai, Mohamad Amr Zaini, Klaas de Lint, Daoud Sie, Martin A. Rooimans, Davy Rockx, Elizabeth E. Hoskins, Najim Ameziane, Rob Wolthuis, Hans Joenje, Susanne I. Wells, Josephine Dorsman

**Affiliations:** 1 Department of Clinical Genetics, Amsterdam UMC, Location VUMC, De Boelelaan 1117, 1118, 1081 HV Amsterdam, The Netherlands; 2 Medpace, Cincinnati, OH 45227 USA; 3 Division of Oncology, Cincinnati Children’s Hospital Medical Center, Cincinnati, OH 45229 USA

Correction to: *Scientific Reports* 10.1038/s41598-021-04042-9, published online 07 January 2022

The original version of this Article contained errors.

Figure 3 and Figure 5 were incorrectly rendered which resulted in incorrect words and symbols appearing in Figure 3B and Figure 5B and 5D.

The original Figure 3 and Figure 5 and their accompanying legends appear below.

**Figure 3:**
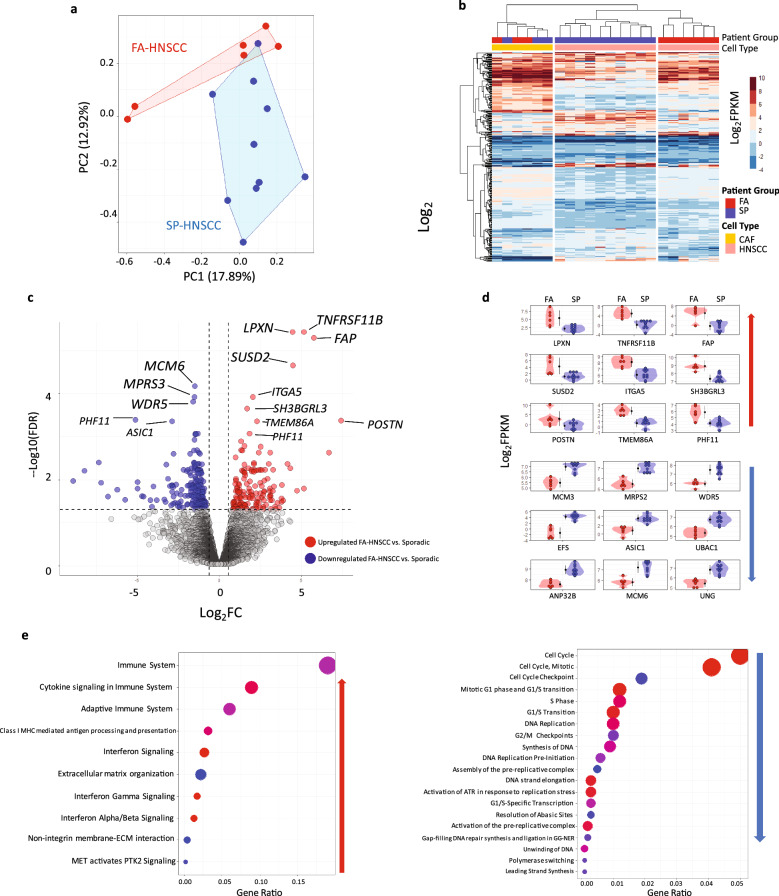
Transcriptomic analysis of FA/SP-HNSCC cell lines. FA-defects confer significant expression differences. (**a**) Principal component analysis (PCA) with Partition Around Medoids (PAM). Based on normalized expression values of all genes with at least minimum expression (CPM > 2). PAM clustering was set at two (k = 2). FA and SP samples were interrelated for the first PC of expression, and they only show a partial overlap according to the second PC. (**b**) Hierarchical clustering with heatmap for 363 differentially expressed genes (DEGs) between FA and SP HNSCCs (FDR < 0.05). Columns represent samples. Rows represent genes. The results reveal distinctions between FA and SP HNSCCs, but not between the cancer-associated FA and SP fibroblasts (CAFs). (**c**) Volcano plot for FA-HNSCC DEGs. (**d**) Violin-Dot plots for top FA-HNSCCs DEGs (FDR < 0.001). The top panel represents FA-HNSCC-upregulated genes. The bottom panel shows the top downregulated genes in FA-HNSCC cells. (**e**) Bubble plots for the significantly differentially expressed pathways in FA-HNSCC compared to SP. Pathway analysis was done with Reactome. Pathways were ranked based on gene ratio (Gene in list/Genes in pathway). FA-HNSCC cells were associated with upregulation of immune response/interferon-signaling associated pathways and downregulation of cell-cycle related pathways compared to SP-HNSCCs. Expression values as normalized Log_2_ FPKM, FA Upregulated (Log_2_ FC > 0, FDR < 0.05), FA downregulated (Log_2_ FC < 0, FDR < 0.05).

**Figure 5:**
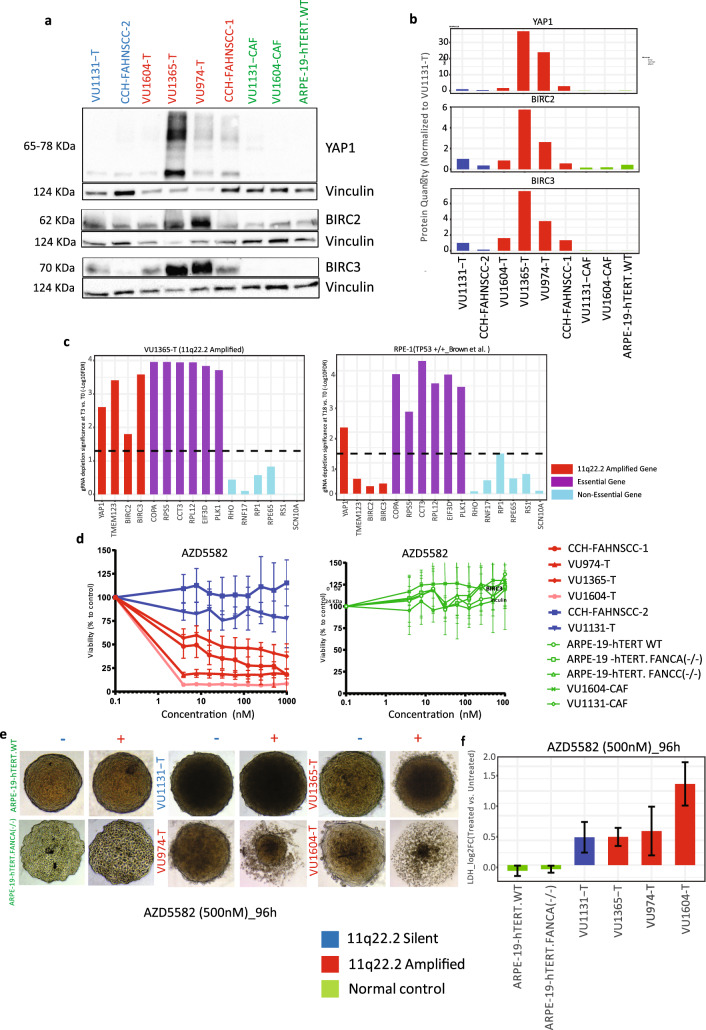
YAP1 and BIRC2-3 functional roles in FA-HNSCC cellular viability. The inhibition of BIRC2-3 selectively inhibits growth of FA-HNSCC cell lines with 11q22.2 amplification and no adverse effects on normal cells. (**a**) YAP1/BIRC2-3 western blots depict protein expression in FA-HNSCCs and a panel of normal controls. Vinculin is utilized as a loading control. YAP1, BIRC2 and BIRC3 proteins are most strongly expressed in the FA-HNSCC panel. (**b**) Bar charts representing YAP1, BIRC2 and BIRC3 protein levels in relation to vinculin and normalized to 11q22.2 silent VU1131-T. FA-HNSCC samples harboring 11q22.2 amplification show overall elevated expression of YAP1 and BIRC3 proteins, compared to 11q22 silent HNSCCs and a panel of normal immortalized cells. (**c**) Bar charts depicts the overall depletion significance (− Log_10_FDR) of gRNAs targeting 11q22.2 amplified genes and a panel of known essential and known non-essential genes in VU1365-T (left) and RPE-1 cells (Right). Depletions with FDR < 0.05 were considered significant. All gRNA targeting the selected 11q22.2-residing genes displayed significant sensitizing effects, in ranges comparable to guides targeting known essential genes. *YAP1* turned out to be the only 11q22.2 amplification gene with its gRNAs significantly depleted in both VU1365-T as well as the normal cell model, suggesting a more general vitality role for *YAP1.* (**d**) Dose response with the BIRC2/BIRC3 inhibitor AZD5582. FA-HNSCC cells are depicted on the left and untransformed cells on the right. AZD5582 treatment for 96 h resulted in significant reduction in viability in FA-HNSCC with 11q22.2 amplification compared to 11q22.2 silent tumors or normal cells. VU974-T and VU1604 exhibit the most dramatic response to the drug treatment and reach IC50 by 3.9 nM. VU1365-T was the most resistant 11q22.2 amplified cell line with AZD5582 and reached IC50 at 250 nM. None of the 11q22.2 silent or normal cells show an adverse negative response to AZD5582 in concentration ranges between 3.8–1000 nM. (**e**) AZD5582 treatment of a panel of FA-HNSCC and normal spheroid cultures. Treatment with AZD5582 (500 nM) for 96 h resulted in cell detachment or spheroid disintegration specifically in 11q22.1 amplified FA-HNSCC cells. (**f**) Lactate dehydrogenase (LDH) cytotoxicity assay. Y axis indicates the Log_2_-transformed fold changes in LDH level between AZD5582-treated and untreated samples.

Additionally, the Acknowledgments and Funding section in the original version of this Article were incomplete.

“We would like to thank KWF (www.kwf.nl/) and CCA for financial support. We would like to thank Yne Waterham and Saskia Van Mil for their lab support. We would like to thank all the colleagues at the Oncogenetics department for their feedback and support.”

now reads:

“We would like to thank the KWF Dutch cancer society (KWF; www.kwf.nl), Stichting Cancer Center Amsterdam (CCA) and the Fanconi Anemia Research Fund (FARF; www.fanconi.org) for financial support. We would like to thank Yne Waterham and Saskia Van Mil for their lab support. We would like to thank all the colleagues at the Oncogenetics department for their feedback and support.”

“Funding is partly supported by Cancer Centre Amsterdam (CCA). KWF.”

now reads:

“Funding is partly supported by the KWF Dutch cancer society (KWF; www.kwf.nl), Stichting Cancer Center Amsterdam (CCA) and the Fanconi Anemia Research Fund (FARF; www.fanconi.org).”

The original Article has been corrected.

