# Supplementary material for: BIRC2–BIRC3 amplification: a potentially druggable feature of a subset of head and neck cancers in patients with Fanconi anemia
Source: Sci Rep. 2022 Jan 7;12:45. doi: 10.1038/s41598-021-04042-9 (PMC8742043; doi:10.1038/s41598-021-04042-9)
Supplement: Supplementary file 1 — Supplementary Information 1. [file 41598_2021_4042_MOESM1_ESM.pdf]

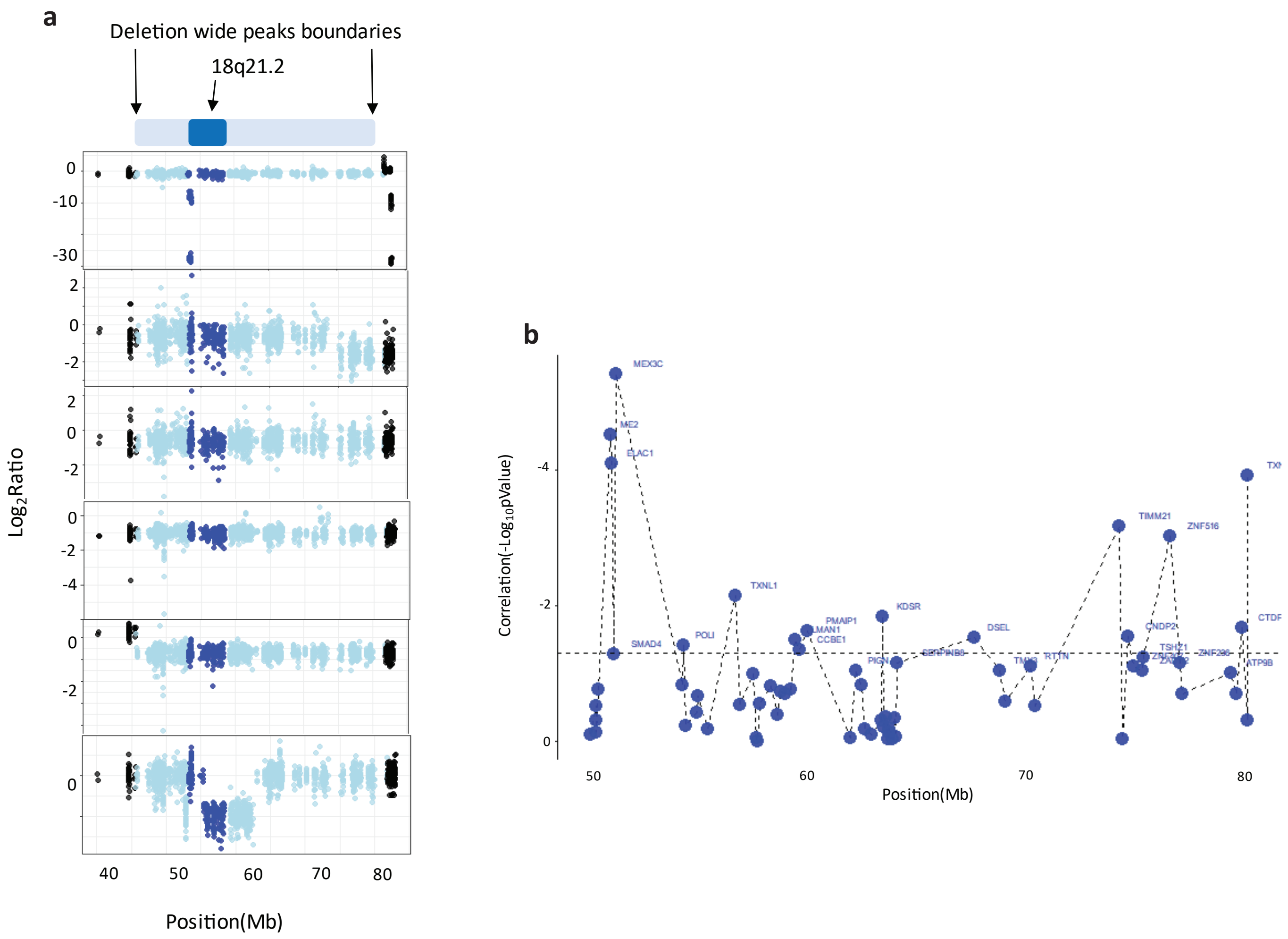

Supp.Figure 1

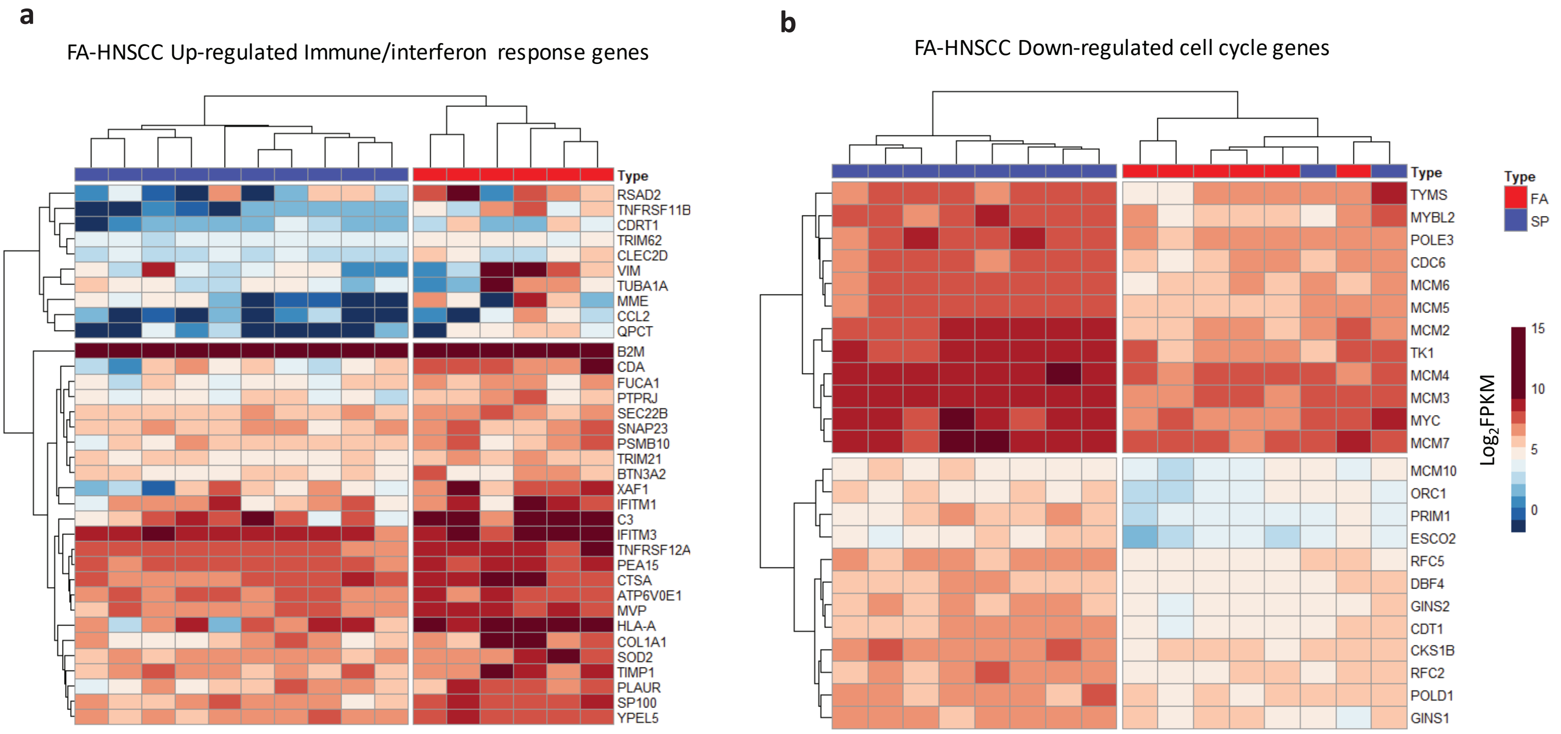

Supp.Figure 2

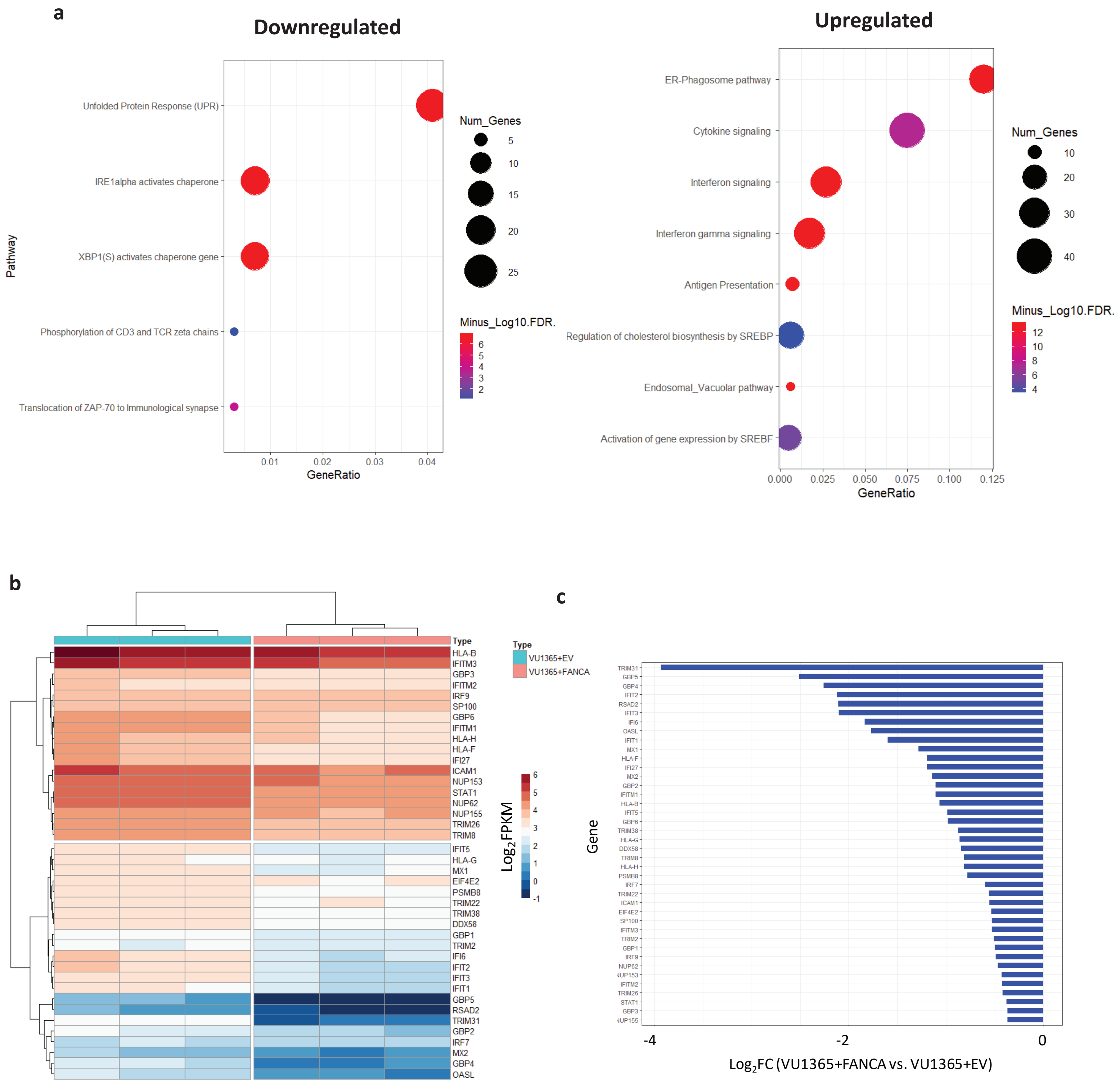

Supp.Figure 3

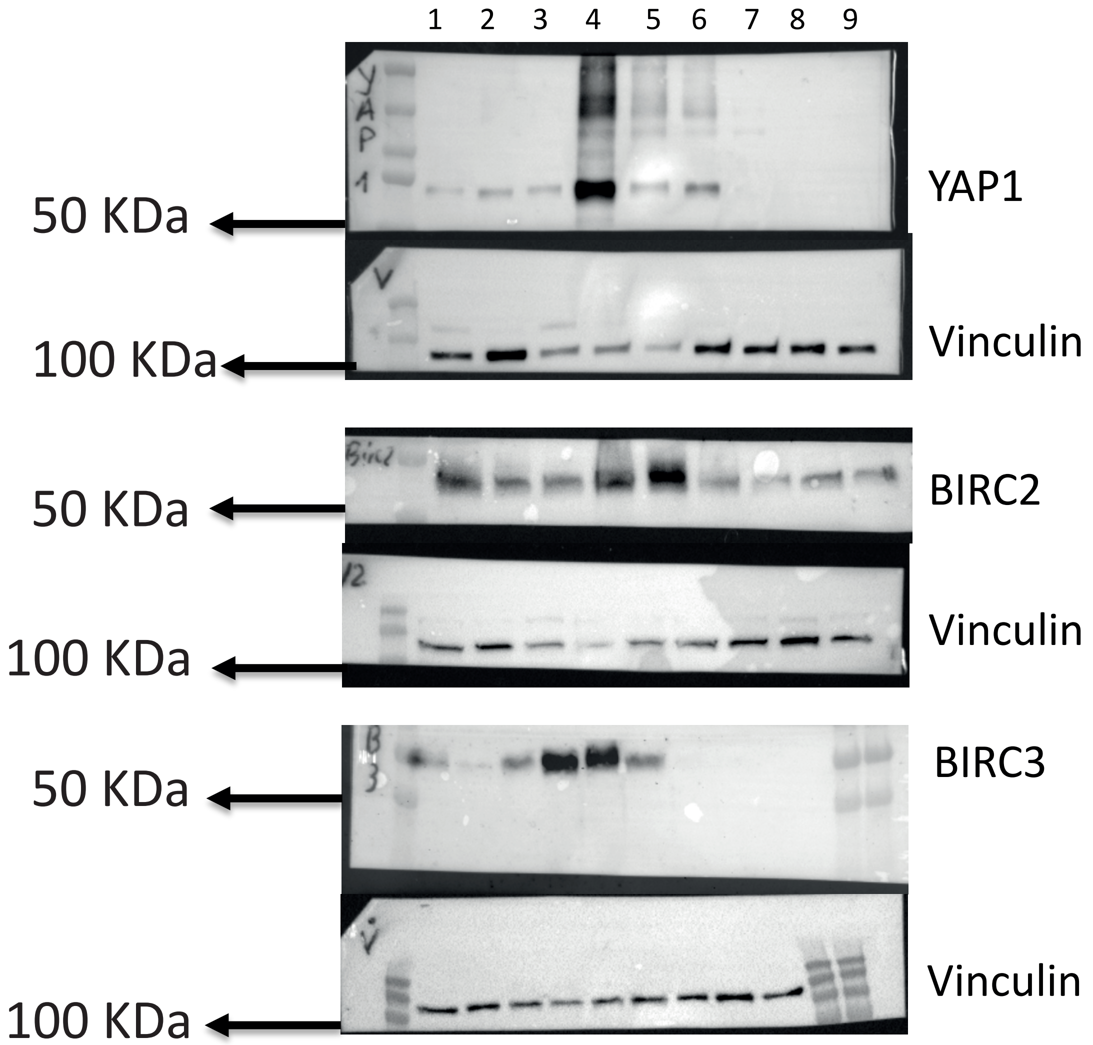

Supp.Figure 4

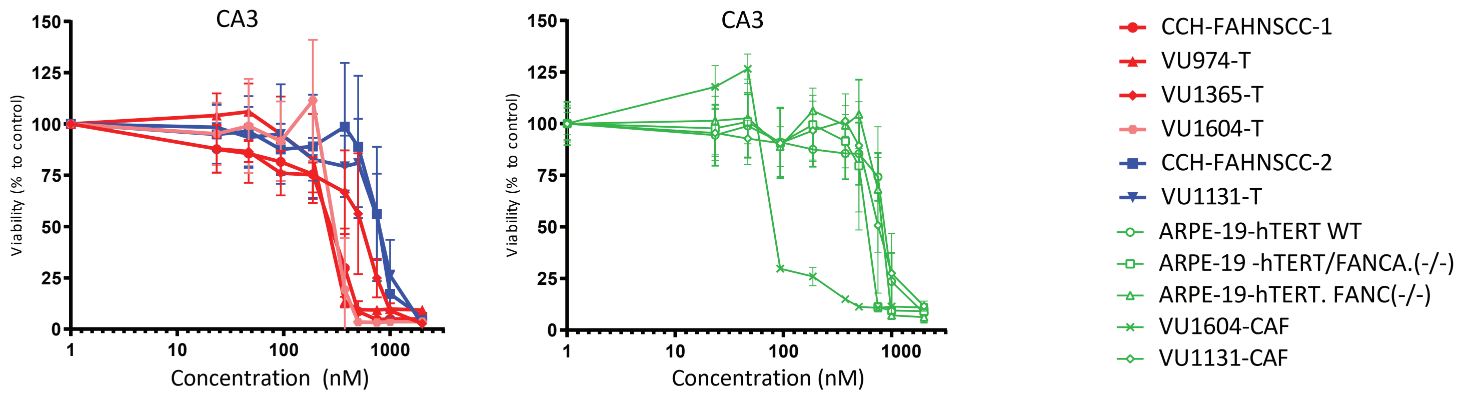

Supp Figure 5

Supplementery.Table.1. TP53 somatic mutations in FA-HNSCC derived cell models

Sample	Ref	Alt	Function	AA.Change.ref.Gene	ExAC	SIFT	cosmic90	Clinvar	AF
VU1604.-T	CA	-	frameshift deletion	TP53:NM_001126115:exon2:c.251_252del:p.V84Gfs*4 TP53:NM_001276699:exon2:c.170_171del:p.V57Gfs*4 TP53:NM_001126118:exon5:c.530_531del:p.V177Gfs*4 TP53:NM_001126114:exon6:c.647_648del:p.V216Gfs* TP53:NM_001276761:exon6:c.530_531del:p.V177Gfs*4	NA	.	No	.	1.00
VU1365-T	G	A	nonsynonymous SNV	TP53:NM_001126115:exon4:c.C448T:p.R150W TP53:NM_001276699:exon4:c.C367T:p.R123W TP53:NM_001126118:exon7:c.C727T:p.R243W TP53:NM_001126114:exon8:c.C844T:p.R282W TP53:NM_001276761:exon8:c.C727T:p.R243W	1.66E-05	D	Yes	CLINSIG=pathogenic	0.500
VU1131-T	C	A	nonsynonymous SNV	TP53:NM_001276699:exon4:c.G341T:p.R114L TP53:NM_001126118:exon7:c.G701T:p.R234L TP53:NM_001126114:exon8:c.G818T:p.R273L TP53:NM_001276695:exon8:c.G701T	8.76E-06	D	Yes	.	1.00
VU974-T	A	-	frameshift deletion	TP53:NM_001126118:exon3:c.6delT:p.D2Efs*2 TP53:NM_001276695:exon4:c.6delT:p.D2Efs*2	NA	.	No	.	1.00

Supplementary. Figure 1. 18q21.2 Deletion in FA-HNSCC cell lines. A) Genomic copy number plot depicts the position and alternation pattern of q21.2 deletion wide peak boundaries on chromosome 18. Y-axis represents copy number Log_2Ratio, X-axis represents genomic coordinates in Mb. B) Genomic point plot for the significance of copy-number-expression correlations on 18q21.2. Y-axis represents correlations minus Log_{10}-transformed pValue. X axis represents chromosomes as well as genomic coordinates in Mb.

Supplementary. Figure 2. The differentially expressed pathways in FA-HNSCC. Hierarchical clustering with heatmap indicating the expression/cluster pattern of differentially expressed pathways. A) FA-HNSCC upregulated immune/interferon response genes show a clear distinction between FA and SP. B) FA-HNSCC downregulated cell cycle associated pathways. Two sporadic HNSCCs cluster within the FA group. Expression values as normalized Log_2FPKM, FA Upregulated ($Log_2FC > 0$, $FDR < 0.05$), FA downregulated ($Log_2FC < 0$, $FDR < 0.05$).

Supplementary. Figure 3. Transcriptomic outcome of the restoration of the *FANCA* core-complex defect in the FA-HNSCC cell line VU1365-T. A) Bubble plots for the significantly differentially-expressed pathways in *FANCA*-corrected VU1365+FANCA compared to its FANCA-deficient counterpart VU1365+EV. Pathway analysis was performed with Reactome. Pathways were ranked based on gene ratio (Genes in list/Genes in pathway). Correction of the *FANCA* defect in VU1365-T cells was associated with upregulation of unfolded protein response/ chaperone genes activation and downregulation of interferon/immune response related signaling pathways. B) Hierarchical clustering with heatmap shows the expression/cluster pattern of the VU1365-T+FANCA downregulated interferon singling genes. C) Bar chart present relative expression change (Log_2FC) of the 32 interferon signaling in genes in VU1365+FANCA vs

VU1365+EV. Expression values are presented as normalized Log_2FPKM; VU1365-T+*FANCA* Upregulated ($Log_2FC > 0$, $FDR < 0.05$), VU1365-T+*FANCA* Downregulated ($Log_2FC < 0$, $FDR < 0.05$).

Supplementary. Figure 4. Western blotting images for YAP1, BIRC2, BIRC3 proteins expression and their corresponding vinculin controls 1.VU1131-T, 2.CCH-FA-HNSCC-2, 3.VU1604-T, 4.VU1365-T, 5.VU974-T, 6.CCH-FA-HNSCC-1, 7.VU1131-CAF, 8.VU1604-CAF, 9.ARPE-19-hTERT.WT.

Supplementary. Figure 5. Dose response assay with the YAP1-TEAD interaction inhibitor CA3. The 11q22.2 amplified samples indicate an overall lower CA3 IC50 compare to the 11q22.2 silent and ARPE-19 samples (350-500 vs 750 nM). However, the matched normal control fibroblasts from VU1604-T exhibit a similar IC50 compared to its tumor counterparts.
